# Using RNA-Seq to Identify Reference Genes of the Transition from Brown to White Adipose Tissue in Goats

**DOI:** 10.3390/ani10091626

**Published:** 2020-09-10

**Authors:** Linjie Wang, Xingyue Chen, Tianzeng Song, Xujia Zhang, Siyuan Zhan, Jiaxue Cao, Tao Zhong, Jiazhong Guo, Li Li, Hongping Zhang, Yan Wang

**Affiliations:** 1 Farm Animal Genetic Resources Exploration and Innovation Key Laboratory of Sichuan Province, College of Animal Science and Technology, Sichuan Agricultural University, Chengdu 611130, China; wanglinjie@sicau.edu.cn (L.W.); mooncrawl@163.com (X.C.); zhangxujia@stu.sicau.edu.cn (X.Z.); siyuanzhan@sicau.edu.cn (S.Z.); jiaxuecao@sicau.edu.cn (J.C.); zhongtao@sicau.edu.cn (T.Z.); jiazhong.guo@sicau.edu.cn (J.G.); lily@sicau.edu.cn (L.L.); zhp@sicau.edu.cn (H.Z.); 2 Institute of Animal Science, Tibet Academy of Agricultural and Animal Husbandry Science, Lhasa 850009, Tibet, China; songtianzeng@china.com.cn

**Keywords:** goat, brown adipose tissue, reference gene, Normfinder, BestKeeper, geNorm

## Abstract

**Simple Summary:**

Brown adipose tissue (BAT) plays important roles in unique non-shivering thermogenesis. It is necessary to select reference genes during the transition process from brown (BAT) to white adipose tissue (WAT) for Quantitative PCR (qPCR) analysis. In this study, *CTNNB*, *PFDN5* and *EIF3M*, selected from RNA sequencing data, were the most suitable reference genes. The present study provides a detailed analysis of the expression stability of reference genes for the study of gene expression profiling during the transition process from BAT to WAT.

**Abstract:**

Brown adipose tissues have unique non-shivering thermogenesis functions, can be found in newborn ruminate animals, and then are gradually replaced by white adipose tissues in adulthood. For the purpose of exploring the intrinsic mechanism underlying the conversion process from brown (BAT) to white adipose tissue (WAT), it is necessary to utilize Quantitative PCR (qPCR) to study gene expression profiling. In this study, we identified reference genes that were consistently expressed during the transformation from goat BAT to WAT using RNA-seq data. Then, twelve genes were evaluated as candidate reference genes for qPCR in goat perirenal adipose tissue using three tools (geNorm, Normfinder, and BestKeeper). In addition, the selected reference genes were used to normalize the gene expression of *PGC-1α* and *GPAT4*. It was found that traditional reference genes, such as *GAPDH*, *RPLP0*, *HPRT1*, and *PPIA* were not suitable for target gene normalization. In contrast, *CTNNB*, *PFDN5*, and *EIF3M*, selected from RNA sequencing data, showed the least variation and were recommended as the best reference genes during the transformation from BAT to WAT.

## 1. Introduction

It is well known that three types of adipose tissues exist in mammals, each with their own unique marker genes, which can also be differentiated from their morphology [1,2]. White adipose tissue (WAT) is stored in a single chamber with large lipid droplets. Brown adipose tissue (BAT) is stored in a multi-chamber with small lipid droplets.compared to BAT [3,4,5]. In addition, they can convert into each other under certain conditions [4,5]. For example, when the experiment subjects are subjected to cold stimulation, adrenaline injection and exercise, white fat has the capacity to take on the characteristics of brown adipocytes [6,7,8]. Similarly, beige and brown fat can be reduced or replaced by white fat due to age, obesity, diabetes and other reasons [4]. In many studies, the three types of adipose tissues are identified, mainly through different functions. White adipose tissue is mainly responsible for energy storage and adipokine excretion. Brown adipose tissue is recognized as a thermogenic organ, and functions to rapidly generate heat to resist cold environments, with the help of UCP1 protein and mitochondria. Then, in particular cases, the beige fat can be induced to develop a thermogenesis function just as well as brown adipocytes [2].

Quantitative PCR (qPCR) technology can quickly and accurately quantify the gene expression at transcriptional levels. The qPCR method has been reported to be more specific and powerful to determine the gene expression at different developmental stages or under different conditions [9,10]. However, the results of qPCR are affected by mRNA integrity and the efficiency of reverse transcription [11]. Therefore, it is necessary to utilize reference genes to standardize the samples’ variation. As a reference gene, it should not be easily influenced by external factors. However, the reference gene expression may change under different types of tissues and different treatment conditions; therefore, it is important to choose appropriate reference genes to minimize these differences across all samples [12,13].

It has been shown that brown adipose tissue is mainly found in the intrascapular, perirenal, and pericardial areas of newborns. With an increase in age, it is gradually replaced by white adipose tissue and adult animals have very little brown adipose tissue [2,14]. In our recent study, we found that BAT mainly distributed in the perirenal fat at 1 day after birth, and there was obviously a disappearance of BAT from 1 day to 1 year after birth by UCP1 immunohistochemistry and qPCR analysis [15]. It has been demonstrated that BAT plays an important role in non-shivering thermogenesis, which is essential for cold adaptation though enhancing energy expenditure [16,17]. In addition, the browning of WAT is induced by certain external factors, such as cold exposure, exercise, and food components [18]. Recent studies have shown that dietary sea buckthorn induces the browning of WAT in lambs, indicating that we can modulate BAT thermogenesis in lambs through nutrition regulation [19]. Consequently, the study of gene expression associated with the transformation from BAT to WAT is essential to identify genes involved in related biological processes. Therefore, it is necessary to select stable reference genes for the normalization of gene expression. In this study, we used RNA-seq data during the transition process from goat BAT to WAT combined with previous studies to select 12 candidate reference genes using geNorm [20], Normfinder [21] and BestKeeper [22] tools. In addition, *PGC-1α* and *GPAT4* were taken as the target genes to correct the stability of candidate reference genes.

## 2. Materials and Methods

### 2.1. Ethics Statement

All research involving animals was conducted according to the approved protocols of the Institutional Animal Care and Use Committee at the College of Animal Science and Technology, Sichuan Agricultural University, Sichuan, China, under permit No. DKY-B20176925.

### 2.2. Animals

In the present study, Chuanzhong black goats were raised at the breeding center of the Sichuan Agricultural University, Ya’an, China (~1000 m altitude; 103.00° E, 29.98° N). The annual average temperature and humidity in Ya’an are about 14 °C and 52%, respectively. The Chuanzhong black goat is one of the most important breeds for meat production in the southwest of China. A detailed description of the experimental design and sample collection was provided in our previous study [15]. The perirenal adipose tissues were collected from 12 female Chuanzhong black goats at three postnatal periods (1 day, 30 days, and 1 year after birth, 4 individuals at each stage, shown as D1, D30, and Y1, respectively). All goats were reared under standard conditions, fed with a diet of hay and concentrate (forage to concentrate ratio, 70:30) twice a day, and were free to access clean water. All animals were separated from their diet for 24 h, and given no water for 12 h before slaughter. Then, perirenal adipose tissues (Figure S1) were collected and were quickly put into liquid nitrogen, and stored at −80 °C.

### 2.3. RNA Extraction and cDNA Synthesis

Total RNA was extracted using RNAiso plus (TaKaRa, Tokyo, Japan). RNA concentration was measured using a NanoDrop 2000 spectrophotometer (Agilent, Santa Clara, CA, USA). The RNA purity was distinguished by the ratio of OD260/280, and RNA quality was evaluated by RIN (RNA Integrity Number) value, which was calculated using the Agilent 2100 Bioanalyzer System (Agilent, Santa Clara, CA, USA). Each sample was reverse transcribed with 1 μg total RNA using the Reverse Transcription Kit (TaKaRa, Tokyo, Japan).

### 2.4. Statistical Analysis of the Reference Genes from RNA-Seq Data

To identify genes whose expression had a low variation during the transformation from goat BAT to WAT, we retrieved 12 RNA-seq datasets previously published in relation to the transformation from BAT to WAT [15]. The raw data are available in the NCBI (National Center for Biotechnology Information) sequence read archive (SRA) under accession number PRJNA547456. The selection standards in this study were the coefficient of variation (CV) < 0.15 and the fragments per kb per million reads (FPKM) values were between 100 and 1000 to make sure the reference genes had a similar expression with detectable levels across samples. 

### 2.5. Use Bio-Rad CFX Real-Time Quantitative Instruments for qPCR

The primer pairs of reference genes were designed by Primer Premier 5.0 software. The information of primers was shown in Table 1. Before qPCR, the specificity and amplification efficiency of the primers were detected by the melting curve and standard curves. Furthermore, all PCR products were sent to BBI Life Sciences Corporation (Shanghai, China) for sequencing to confirm the amplification of target genes. The reaction volume was 10 µL, containing 5 μL SYBR (Synergy Brands) Green Real Time PCR Master Mixture (Takara, Tokyo, Japan), 0.8 µL of primers, and 0.8 µL of cDNA. The qPCR was launched with an initial step at 95 °C (3 min) followed by 40 cycles of 20 s at 95 °C for DNA denaturation and 30 s at 60 or 61.3 for annealing at and 20 s at 72 °C for elongation, and a final extension for 5 min.

### 2.6. Analysis of the Expression Stability for Candidate Reference Genes

The gene expression was detected by Bio-Rad CFX 96 (Bio-Rad, Hercules, CA, USA). Then, the data were imported into geNorm, NormFinder, and BestKeeper, as required by each program and according to their manuals. Thus, the delta-Ct method was used to transfer Ct values to linear-scale expression quantities. The delta-Ct formula is as follows: Q = E ^delta-Ct^ [delta-Ct = min-Ct − sample-Ct, E = amplification efficiency (2 = 100%), min-Ct = lowest Ct value = Ct value of sample with highest expression]. Moreover, the data processing on BestKeeper was based on raw Ct-values from qPCR.

The highest M value of the reference gene in geNorm is considered to be the most stable gene. A pairwise variation V value below 0.15 indicates that the inclusion of an additional reference gene is not needed. The algorithm of NormFinder estimates the overall expression variation and the variation between subgroups of the sample set. In addition, the lowest stability value of the reference gene is considered to be the most stable gene in the sample set investigated. In the BestKeeper tool, pairwise correlations and regression analysis are used as process approaches. The highest stability reference gene has the lowest values for the coefficient of variation (CV) and standard deviation (SD), but has the highest correlation coefficient (*r*) value.

### 2.7. Validation of Selected Reference Genes

Target genes were chosen for validating the stability of the reference gene by comparing their expression patterns after normalization. *PGC-1α* and *GPAT4* were selected as target genes because they are the marker genes for brown adipose tissue. Finally, the expression levels of *PGC-1α* and *GPAT4* were normalized using the 2^−ΔΔCT^ method [23]. All results were evaluated using one-way ANOVA and Duncan’s new multiple range test to analyze their statistical significance by GraphPad Prism 6.01.

## 3. Results

### 3.1. Reference Gene Selection during the Transformation from Goat BAT to WAT Using RNA-Seq Data

Based on the FPKM value and the coefficient of variation, we obtained a total of 54 candidate reference genes (Table S1). KEGG (Kyoto Encyclopedia of Genes and Genomes) enrichment analysis indicated that most candidate reference genes were involved in the ribosome and lysosome pathways. Furthermore, four reference genes (*CTNNB1*, *PFDN5*, *RPL22*, and *EIF3M*) were selected for further validation. Among them, *CTNNB1* and *PFDN5* genes have the lowest coefficient of variation. In addition, we selected four reference genes (*TBP*, *PPIA*, *HPRT*, and *RPLP0*) according to previous studies in white and brown adipose tissue [24,25,26]. Four traditional reference genes were also used, including *GAPDH*, *18SrRNA*, *YWHAZ*, and *ACTB* genes [27,28,29].

### 3.2. RNA Purity, Primer Verification and Amplification Efficiency

In this study, the OD260/280 ratio of RNA ranged from 1.81 to 2.12 and the RIN values of all samples ranged from 8.1 to 9.1 (Table S2 and Figure S2), indicating that the RNA was of good purity. These results indicated that total RNA showed no degradation and could be used for the next steps. Agarose gel electrophoresis was carried out for detecting the specificity of the primers of reference genes (Figure S3), and then the fragments were sequenced to verify the reference genes. Furthermore, a single specific amplification melting curve was present. The amplification efficiencies ranged between 90% and 100% (Figure S4).

### 3.3. Expression Stability of the Reference Genes by geNorm Analysis

**Figure 1:**
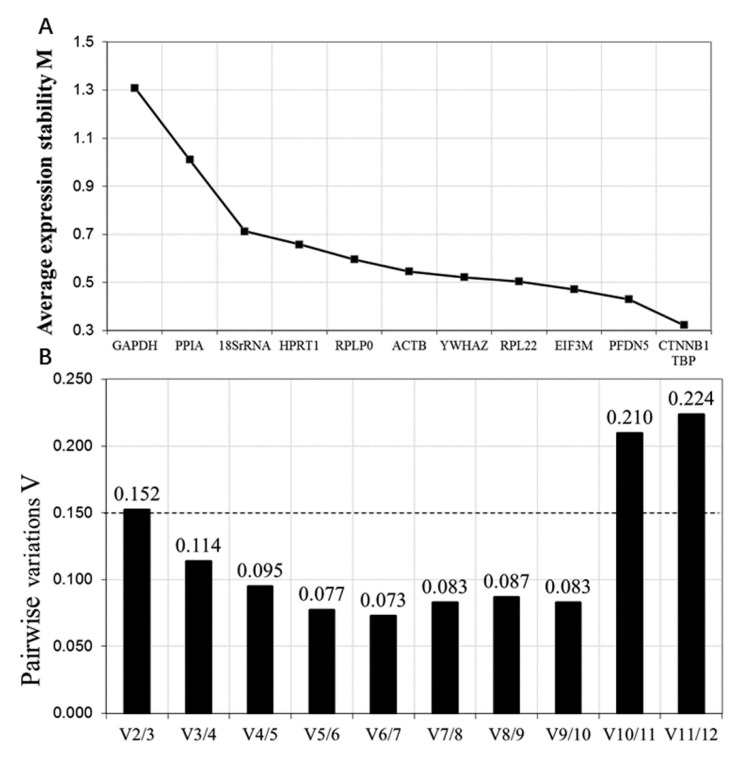
geNorm analysis of selected 12 reference genes. (**A**) The average expression stability measure of candidate reference genes. (**B**) The pairwise variation in 12 reference genes for the determination of the number of candidate reference genes.

geNorm calculated the gene expression stability (M Value) by comparing a specific gene with all of the other reference genes and the average pairwise variation was used to select the optimal number of reference genes. The reference gene with the smallest M value had the strongest stability. As shown in Figure 1A, *CTNNB*, *TBP*, *PFDN5*, and *EIF3M* expressed good stability (M < 0.5), followed by *RPL22*, *YWHAZ*, *ACTB, RPLP0*, and *HPRT1*. The most variable reference genes were *18SrRNA, PPIA* and *GAPDH*. Interestingly, three novel reference genes (*CTNNB*, *PFDN5*, and *EIF3M*) that were selected from RNA-Seq data mostly expressed good stability. In the pairwise variation analysis, we found that the optimal number of reference genes for normalization was three, using 0.15 as a cutoff value (Figure 1B).

### 3.4. Expression Stability of the Reference Genes by NormFinder Analysis

NormFinder calculates the intragroup and intergroup variations. As shown in Table 2, *PFDN5*, *EIF3M*, *CTNNB1*, *TBP*, and *ACTB* were the most stable genes, with stability values less than 0.15. Meanwhile, *YWHAZ*, *18SrRNA*, and *RPL22* showed a relatively lower stability. However, *RPLP0*, *HPRT1*, *GAPDH*, and *PPIA* were the least stable reference genes.

### 3.5. Expression Stability of the Reference Genes by BestKeeper Analysis

BestKeeper software can only input reference genes within 10 numbers to calculate the correlation coefficient (*r*), standard deviation (SD) and coefficient of variation (CV). So, the two least stable reference genes in geNorm were excluded. Firstly, the candidate genes have an acceptable overall variation to be considered reference genes (SD < 1). Secondly, a coefficient of correlation (*r*) calculation was performed. Then, the stability was established based on the coefficient of correlation (*r*). The stability was mainly judged by the correlation coefficient (*r*), then by considering the standard deviation (SD). Thus, *TBP*, *CTNNB1*, and *18SrRNA* were the most stable reference genes as *r* > 0.8, then *PFDN5*, *EIF3M*, and *ACTB* followed, as their *r* > 0.7. The correlation coefficient (*r*) value of *YWHAZ*, *RPLP0*, *HPRT1*, and *RPL22* was less than 0.7, expressed by the bad stability in the results of BestKeeper (Table 3).

### 3.6. Correcting the Stability of Candidate Reference Genes with Target Genes

**Figure 2:**
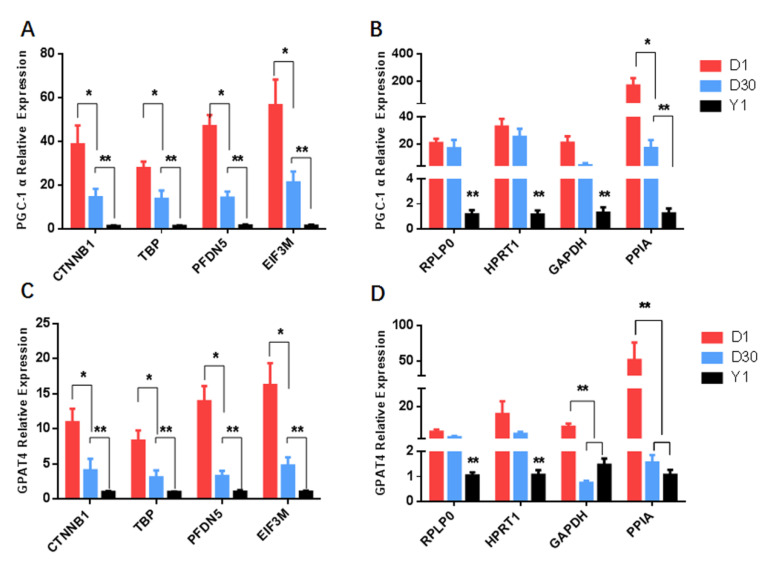
Relative expression of *PGC1α* and *GPAT4* normalized to the different reference genes across three development stages (D1, D30, and YI). (**A**) Relative expression of *PGC1α* by the most stable genes (*CTNNB1*, *TBP*, *PFDN5*, and *EIF3M*). (**B**) Relative expression of *PGC1α* by the least stable reference genes (*RPLP0*, *HPRT1*, *GAPDH*, and *PPIA*). (**C**) Relative expression of *GPAT4* by the most stable genes (*CTNNB1*, *TBP*, *PFDN5*, and *EIF3M*). (**D**) Relative expression of *GPAT4* by the least stable reference genes (*RPLP0*, *HPRT1*, *GAPDH*, and *PPIA*). * *p* < 0.05, ** *p* < 0.01.

To further validate the selection of candidate reference genes, we used either the most (*CTNNB1*, *TBP*, *PFDN5*, and *EIF3M*) or the least stable reference genes (*RPLP0*, *GAPDH*, *HPRT1*, and *PPIA*) to normalize the same target genes. Using *CTNNB1*, *TBP*, *PFDN5*, and *EIF3M* for normalization, the results showed that *PGC-1α* and *GPAT4* were expressed in perirenal adipose tissues with the highest level at D1, and significantly (*p* < 0.05) decreased at D30, then significantly (*p* < 0.01) reached the lowest level at Y1 (Figure 2A,C). When data were normalized to *RPLP0* and *HPRT1*, there was no significant difference between D1 and D30 of *PGC-1α* and *GPAT4* expression (Figure 2B,D). In addition, when using *GAPDH* and *PPIA* as reference genes to normalize *GPAT4*, there was no significant change between D30 and Y1 (Figure 2D).

## 4. Discussion

Brown adipose tissue (BAT) plays important roles in unique non-shivering thermogenesis and is enriched with mitochondria [30]. Although several studies have been conducted to determine the stability of reference genes in adipose tissues, the reference genes for the transformation from BAT to WAT have not been identified. In this study, we used RNA-seq data to select the most suitable reference genes during the transformation from BAT to WAT. Our results showed that *CTNNB*, *TBP, PFDN5* and *EIF3M* genes were the best candidate reference genes. Among them, *CTNNB*, *PFDN5,* and *EIF3M* were selected from RNA sequencing data. In addition, our data indicated that the *TBP* gene expressed good stability. This result is consistent with a previous report that *TBP* is the most stable reference gene between BAT and WAT in mice [31].

In addition, the three tools used in this study have different algorithms, so the stability sorting results of candidate reference genes need to be analyzed synthetically. In geNorm, in calculating the M value of a reference gene, the tool mainly focuses on the comparison between the reference genes, and only the differences between groups are taken into account [20]. In NormFinder, when calculating the stability value of reference gene, the intergroup and intragroup variations are considered, which could reduce the effect of the synergistic genes [21]. BestKeeper is used to evaluate the candidate genes by considering the reference and target genes at the same time, combined with the standard deviation, coefficient of variation, and correlation coefficient [22].

In our results, *ACTB* showed medium stability; however, *ACTB* was quoted as the least stable reference gene during 3T3-L1 adipocyte differentiation [32]. *GAPDH* has been confirmed to have an unstable expression in mesenchymal stem cells during differentiation [28] and shows a variable expression between the white and brown adipose of mice [31]. *PPIA* is the most variable in all stages during 3T3-L1 adipocyte differentiation [25] and is very variable in human perirenal adipose tissues [26], while it proved to be stable when expressed in adipose tissue samples among chow, high-fat, and high-sugar-diet mice [25]. However, *PPIA* is expressed with high stability among different goat tissues (adipose tissue, mammary gland, and liver) [33]. In this study, *GAPDH* and *PPIA* were both proven to be unstable candidate genes by geNorm and NormFinder, respectively. *RPLP0* is the least stable gene in bovine (subcutaneous) and goat (omental) adipose tissues in three physiological states (growing, lactating, and dry) [33], while it has a relatively good stability in the intramuscular fat deposition of skeletal muscles in goats [34]. However, in this study, it was a medium-stability reference gene in goat perirenal adipose tissue.

Previous studies have shown that *PGC-1α* and *GPAT4* genes are the marker genes for brown adipose tissue [30]. In addition, they were differentially expressed in our RNA-seq data (data not shown) during the transition process from brown to white goat adipose tissues. Therefore, *PGC-1α* and *GPAT4* genes were selected as target genes for normalization. *PGC-1α* is the first transcriptional regulatory factor for regulating brown adipose thermogenesis [7,35]. GPAT4 can use exogenous fatty acids for the synthesis of triglycerides to reduce the beta-oxidation in brown adipose tissue [36]. In this study, we found that the patterns of *PGC-1α* and *GPAT4* were, as expected, significantly downregulated in their expression from D1 to Y1, when the two target genes were normalized to *CTNNB1*, *TBP*, *PFDN5,* and *EIF3M*, the four best stability reference genes. However, severe variance appeared when they were normalized to *RPLP0*, *GAPDH*, *HPRT1*, and *PPIA*, the four least stable candidate genes. Hence, for the accuracy of qPCR, appropriate reference genes should be carefully selected, because the unstable reference genes will cause the misinterpretation of expression data.

## 5. Conclusions

In summary, we found that *CTNNB1*, *TBP, PFDN5*, and *EIF3M* were recommended as the most suitable reference genes in qPCR experiments during the transition process from brown to white adipose tissues.

## Figures and Tables

**Table 1:** Primer sequence information used in this study.

Gene Symbol	GenBank No.	Sequence 5′–3′	Tm(°C)	Size (bp)	Eff (%)
PPIA	XM_018047035.1	AAGTCCCGAAGACAGCAGAA	60	209	90.8
		GATGCCAGGACCTGTATGCT			
GAPDH	XM_005680968.3	GCAAGTTCCACGGCACAG	61.3	249	95.7
		GGTTCACGCCCATCACAA			
18S rRNA	DQ149973	TAATCCCGCCGAACCCCATT	61.3	125	93.3
		GGTGTGTACAAAGGGCAGG			
YWHAZ	XM_018058314.1	ACTACTATCGCTACTTGGCTGAG	61.3	84	99.1
		CTTCTTGTTATGCTTGCTGTGA			
ACTB	XM_018039831.1	CCTGCGGCATTCACGAAACTAC	61.3	87	97.4
		ACAGCACCGTGTTGGCGTAGAG			
TBP	XM_018053502.1	TCGCCAAGAATAGTGTGCTG	61.3	202	95.7
		CCGTAAGGCATCATTGGACT			
HPRT1	XM_012167243.2	CGAGATGTGATGAAGGAGATGG	60	186	96.3
		GCCTGTTGACTGGTCGTTAC			
EIF3M	XM_018059285.1	CTGTGCGAGAAACTGGTCAA	60	164	95.7
		ATATACTGGATGGCCCCACA			
PFDN5	XM_005679909.1	GCTTATTGACGTGGGAACT	60	120	98.1
		TGCAGAGCTGGCTGGATT			
CTNNB1	XM_018066894.1	CACAGTTCGATGCTGCTCAT	61.3	161	99.3
		CTGGTCTTCGTCATTCAGCA			
RPLP0	XM_005709526	TTCTCCTTCGGGCTGGTCA	60	104	94.5
		TCCAGGAAGCGGGAATGC			
RPL22	XM_005690753.3	CGGTGTTGTAACAATCGA	60	209	91.9
		CCTCATCTTCCTCCTCTTC			
GPAT4	XM_018041983.1	GGAGTCTCCTTTGGTATCCG	61.4	128	96.8
		CCATTGGTGTAGGGCTTGTA			
PGC-1α	NM_001285631.1	TAAAGCCAACCAAGATAACCC	61.4	242	92.2
		CACCAAACAGCCGAAGACT			

**Table 2:** Expression stability of reference genes by NormFinder analysis.

Gene Name	Stability Value	Rank Order
*PFDN5*	0.13	1
*EIF3M*	0.14	2
*CTNNB1*	0.14	3
*TBP*	0.14	4
*ACTB*	0.14	5
*YWHAZ*	0.16	6
*18SrRNA*	0.18	7
*RPL22*	0.19	8
*RPLP0*	0.21	9
*HPRT1*	0.21	10
*GAPDH*	0.23	11
*PPIA*	0.23	12

**Table 3:** Expression stability of reference genes by BestKeeper analysis.

Gene Name	Coeff. of Corr. [r]	Std Dev [±CT]	CV [% CT]	Rank Order
*TBP*	0.90	0.52	2.22	1
*CTNNB1*	0.83	0.51	2.52	2
*18SrRNA*	0.81	0.97	3.69	3
*PFDN5*	0.78	0.46	2.65	4
*EIF3M*	0.78	0.50	2.51	5
*ACTB*	0.74	0.47	2.60	6
*YWHAZ*	0.66	0.41	2.05	7
*RPLP0*	0.61	0.58	3.51	8
*HPRT1*	0.51	0.54	2.39	9
*RPL22*	−0.01	0.33	1.18	10

## Supplementary Materials

The following are available online at https://www.mdpi.com/2076-2615/10/9/1626/s1. Table S1: Evaluation of selected reference genes using RNA-Seq data. Table S2: Results of sample RNA quality determination. Figure S1: The location of perirenal fat sampling at three stages. Figure S2: RNA integrity analysis using capillary electrophoresis. LM (standard sample), 5S, 18S and 28S ribosomal RNA peaks are denoted. Figure S3: Agarose gel electrophoresis for reference genes. Figure S4: Standard curves of all the reference genes.
