# Supplementary material for: Using RNA-Seq to Identify Reference Genes of the Transition from Brown to White Adipose Tissue in Goats
Source: Animals (Basel). 2020 Sep 10;10(9):1626. doi: 10.3390/ani10091626 (PMC7552189; doi:10.3390/ani10091626)
Supplement: Supplementary file 1 [file animals-10-01626-s001.zip › Additional files/Figure S4.pdf]

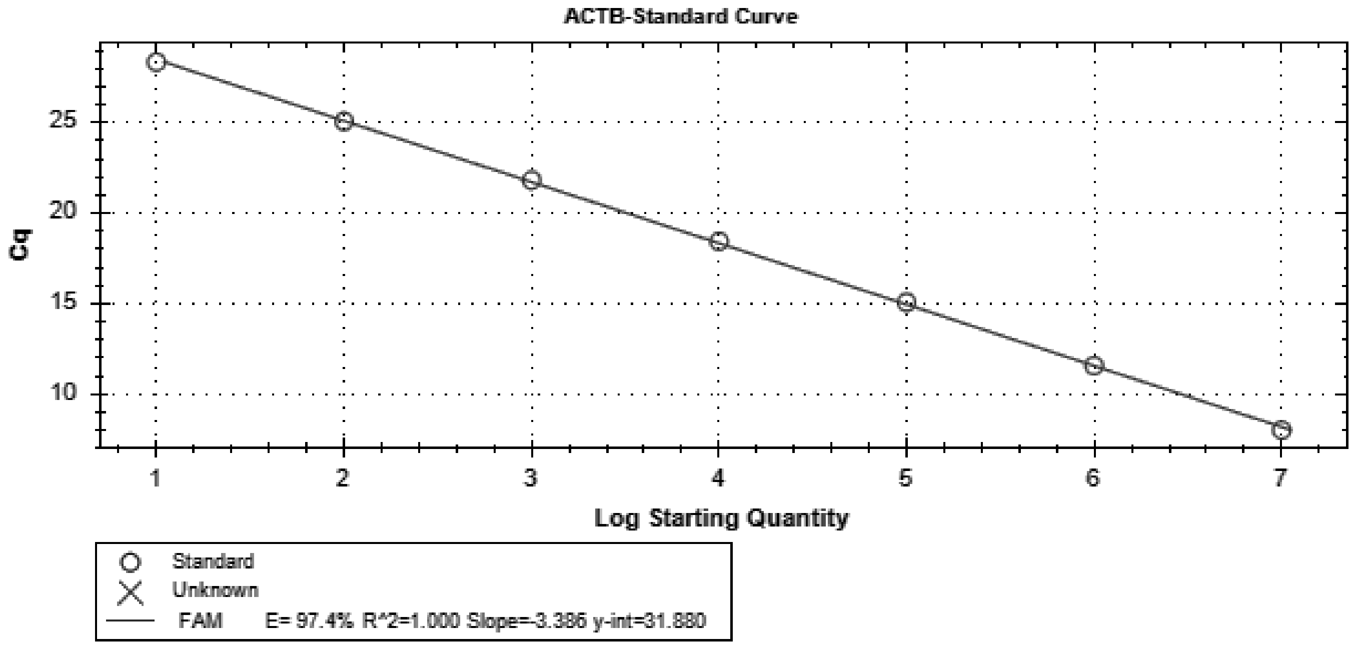

ACTB-Standard Curve
Cq
25
20
15
10
1
2
3
4
5
6
7
Log Starting Quantity
Standard
Unknown
FAM E= 97.4% R^2=1.000 Slope=-3.386 y-int=31.880

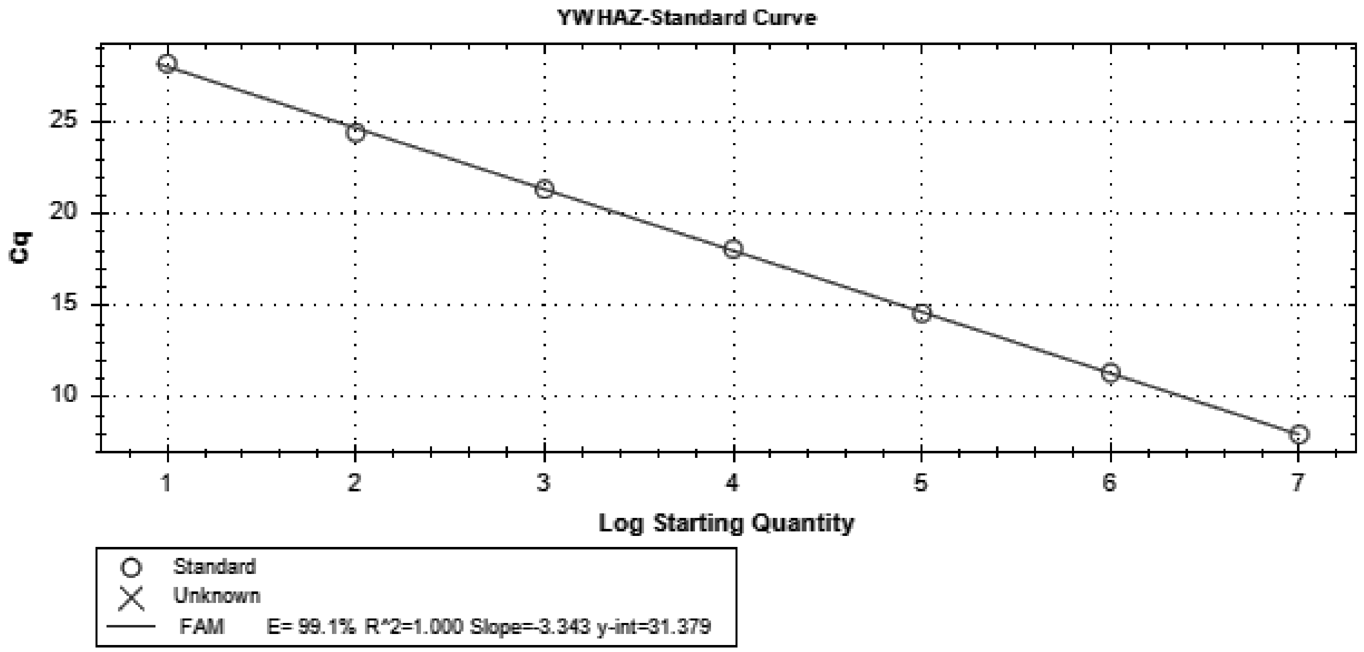

YWHAZ-Standard Curve
Cq
25
20
15
10
1
2
3
4
5
6
7
Log Starting Quantity
Standard
Unknown
FAM E= 99.1% R^2=1.000 Slope=-3.343 y-int=31.379

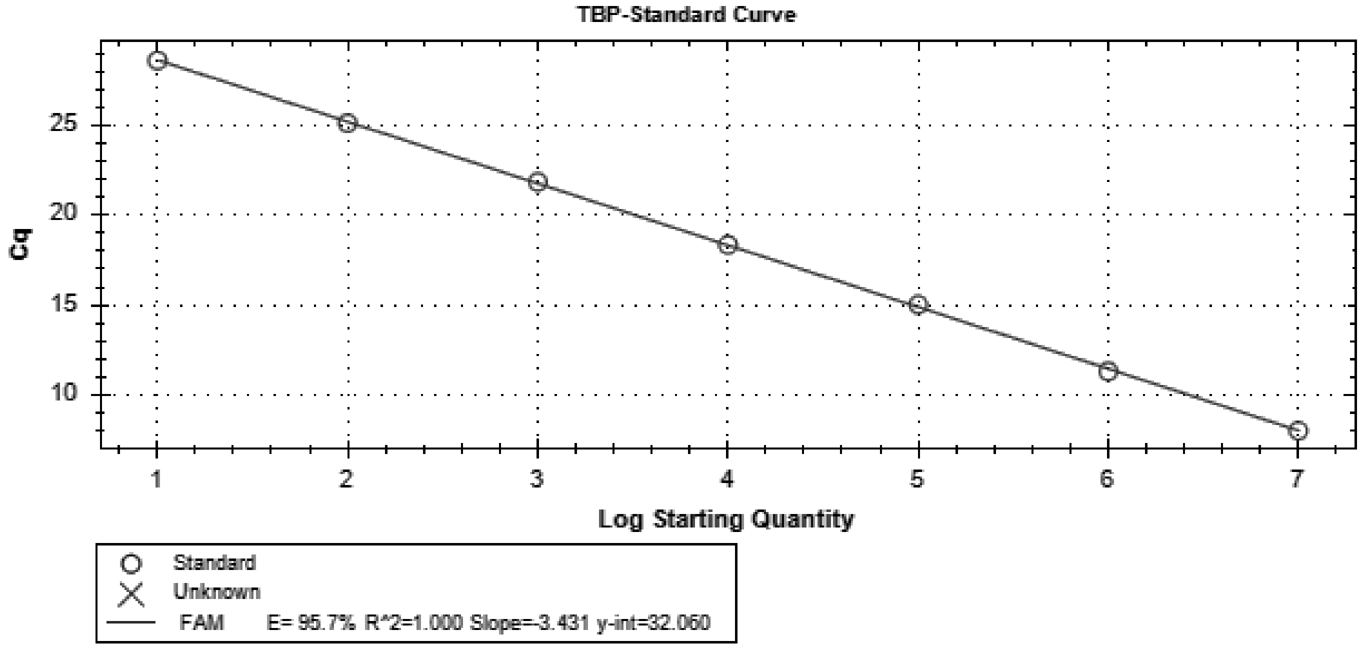
TBP-Standard Curve
Cq
Log Starting Quantity
Standard
Unknown
FAM E= 95.7% R^2=1.000 Slope=-3.431 y-int=32.060

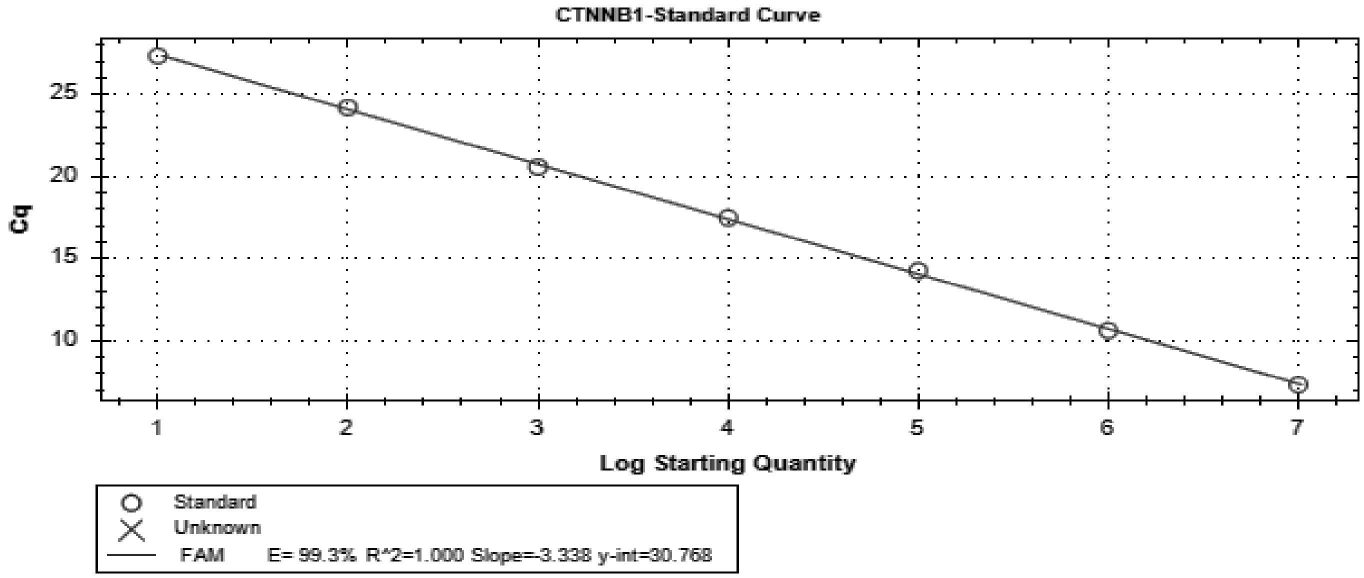
CTNNB1-Standard Curve
Cq
Log Starting Quantity
Standard
Unknown
FAM E= 99.3% R^2=1.000 Slope=-3.338 y-int=30.768

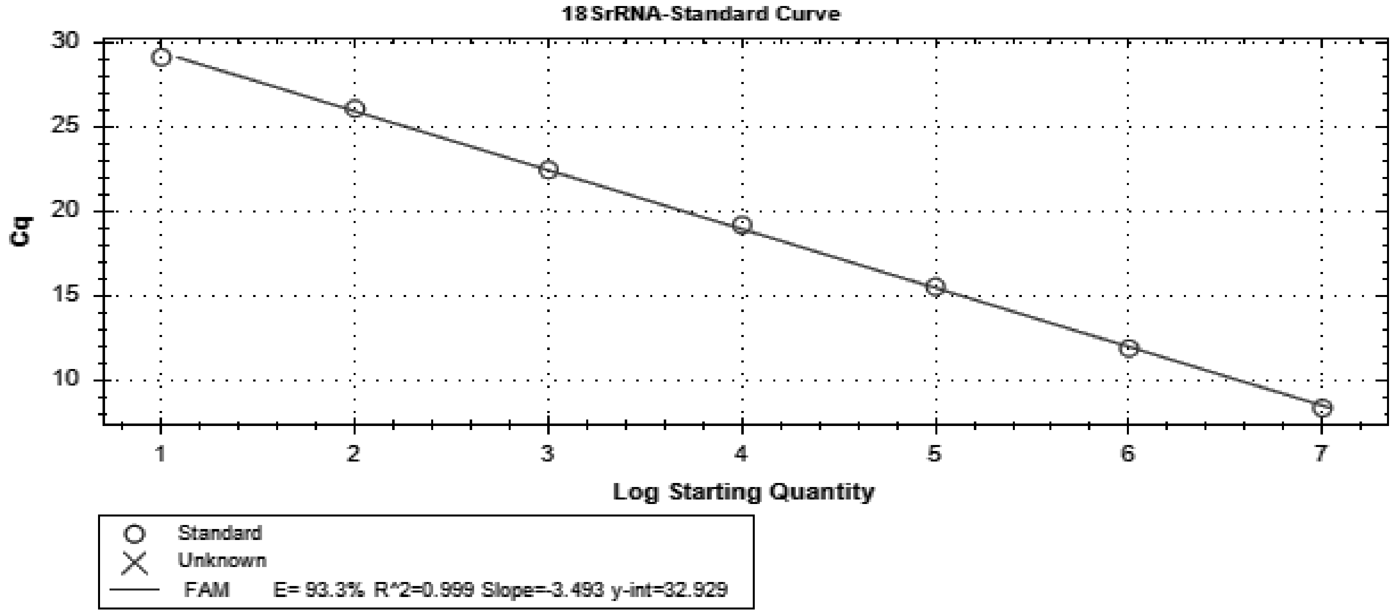
18SrRNA-Standard Curve
Cq
Log Starting Quantity
Standard
Unknown
FAM E= 93.3% R^2=0.999 Slope=-3.493 y-int=32.929

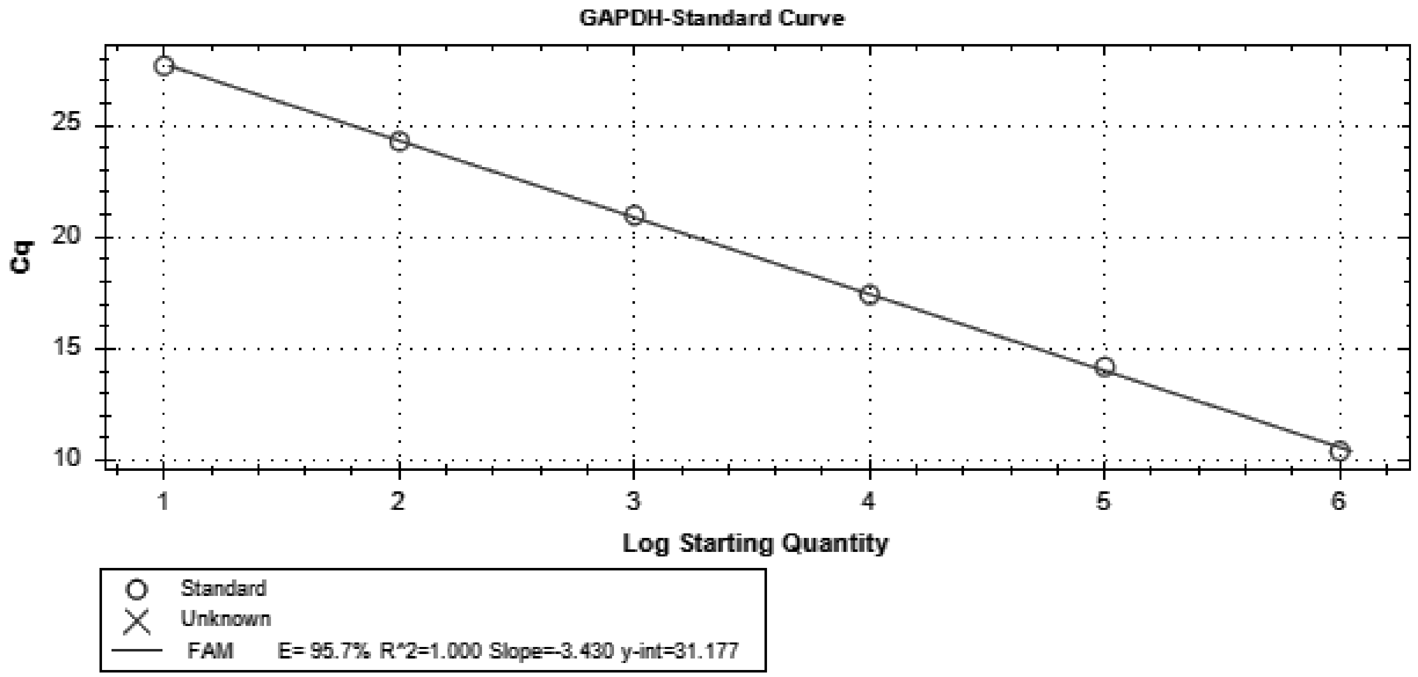

GAPDH-Standard Curve
Cq
Log Starting Quantity
Standard
Unknown
FAM E= 95.7% R^2=1.000 Slope=-3.430 y-int=31.177

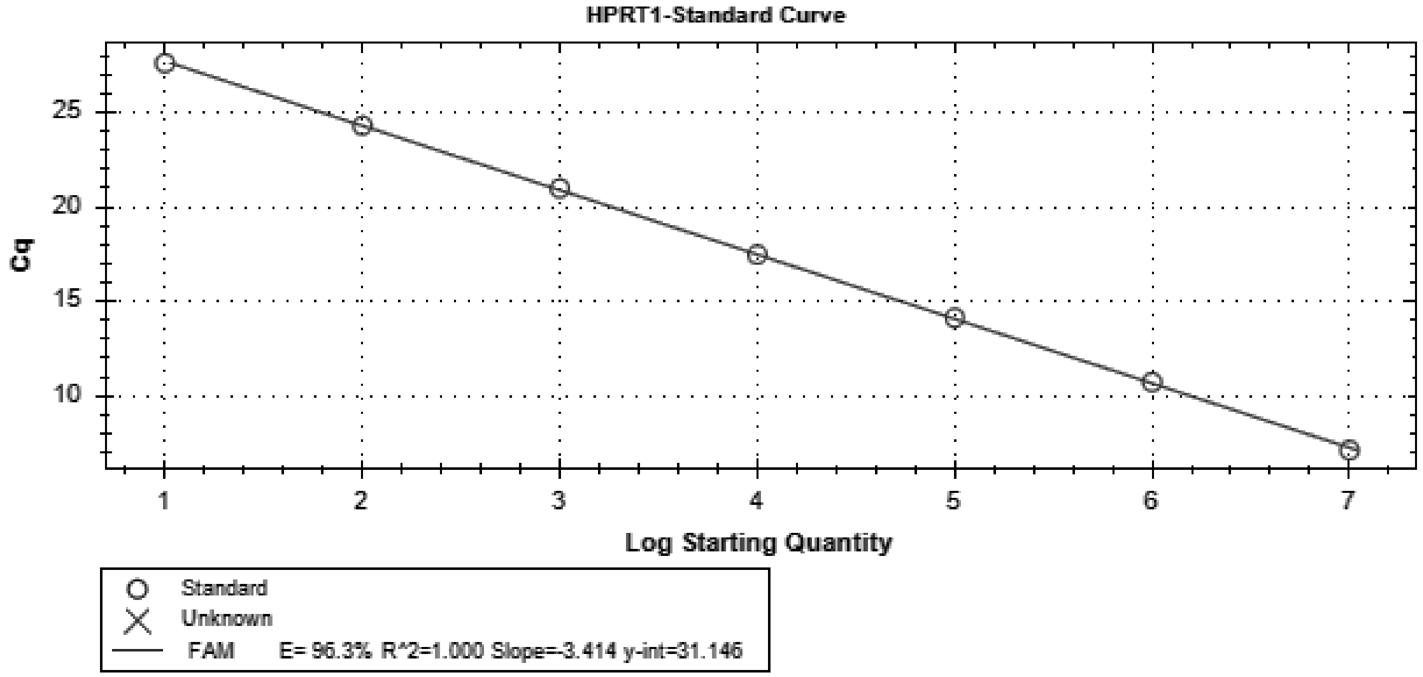

HPRT1-Standard Curve
Cq
Log Starting Quantity
Standard
Unknown
FAM E= 96.3% R^2=1.000 Slope=-3.414 y-int=31.146

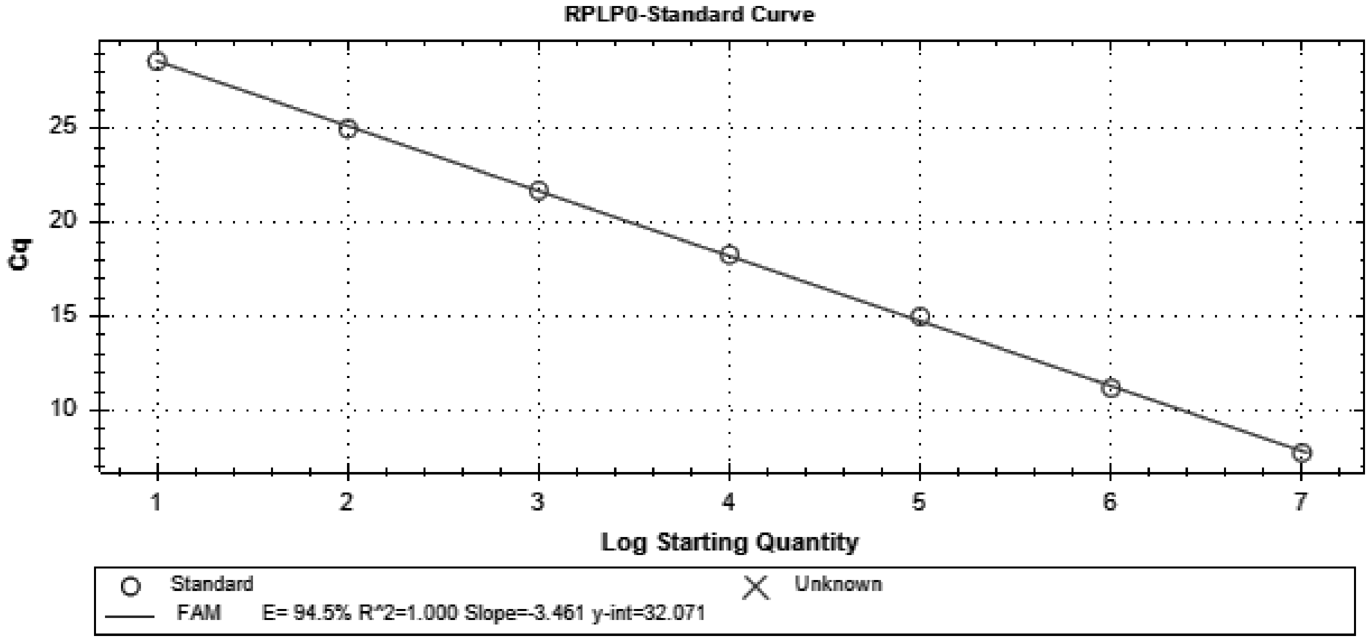
RPLP0-Standard Curve
Cq
25
20
15
10
1
2
3
4
5
6
7
Log Starting Quantity
Standard
Unknown
FAM E= 94.5% R^2=1.000 Slope=-3.461 y-int=32.071

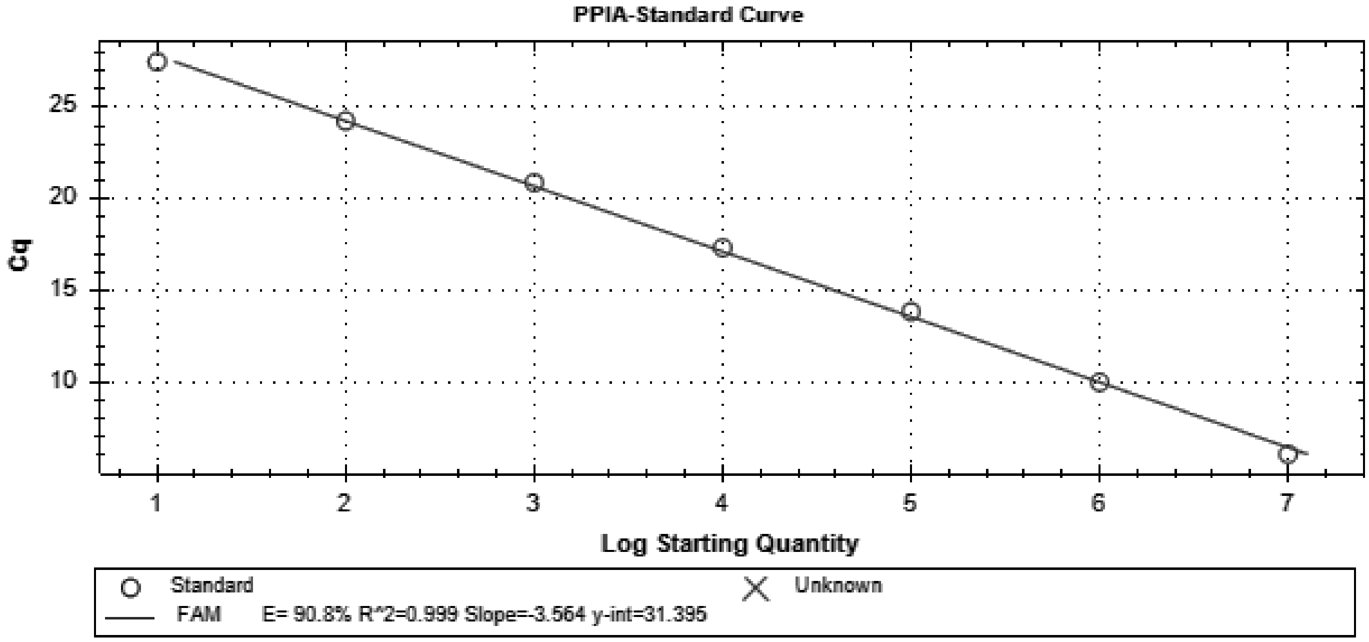
PPIA-Standard Curve
Cq
25
20
15
10
1
2
3
4
5
6
7
Log Starting Quantity
Standard
Unknown
FAM E= 90.8% R^2=0.999 Slope=-3.564 y-int=31.395

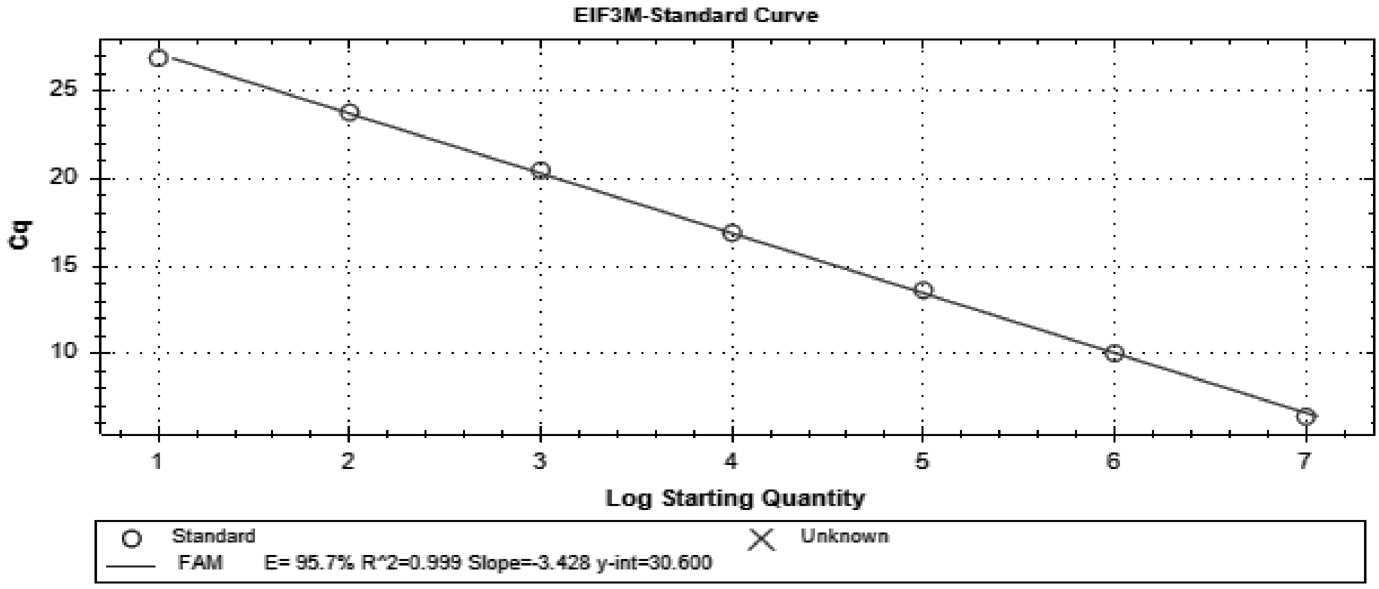
EIF3M-Standard Curve
Cq
25
20
15
10
1
2
3
4
5
6
7
Log Starting Quantity
Standard
Unknown
FAM E= 95.7% R^2=0.999 Slope=-3.428 y-int=30.600

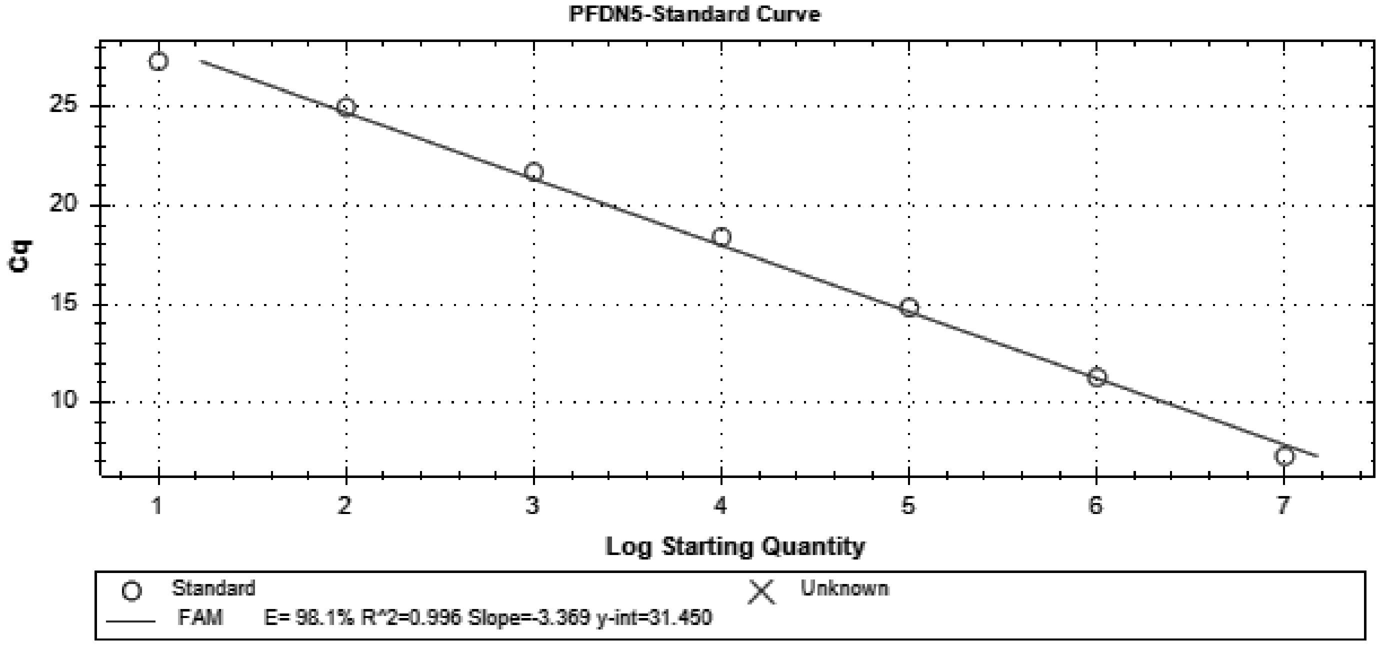
PFDN5-Standard Curve
Cq
Log Starting Quantity
Standard
Unknown
FAM E= 98.1% R^2=0.996 Slope=-3.369 y-int=31.450

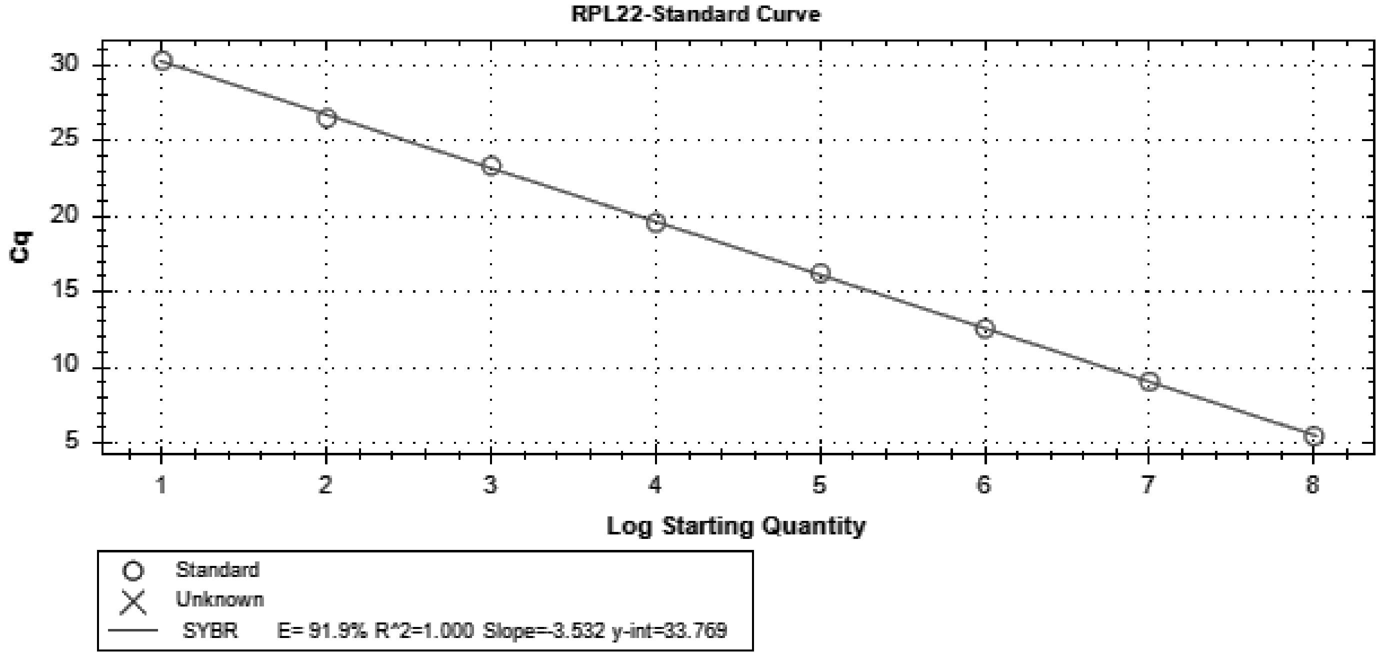
RPL22-Standard Curve
Cq
Log Starting Quantity
Standard
Unknown
SYBR E= 91.9% R^2=1.000 Slope=-3.532 y-int=33.769

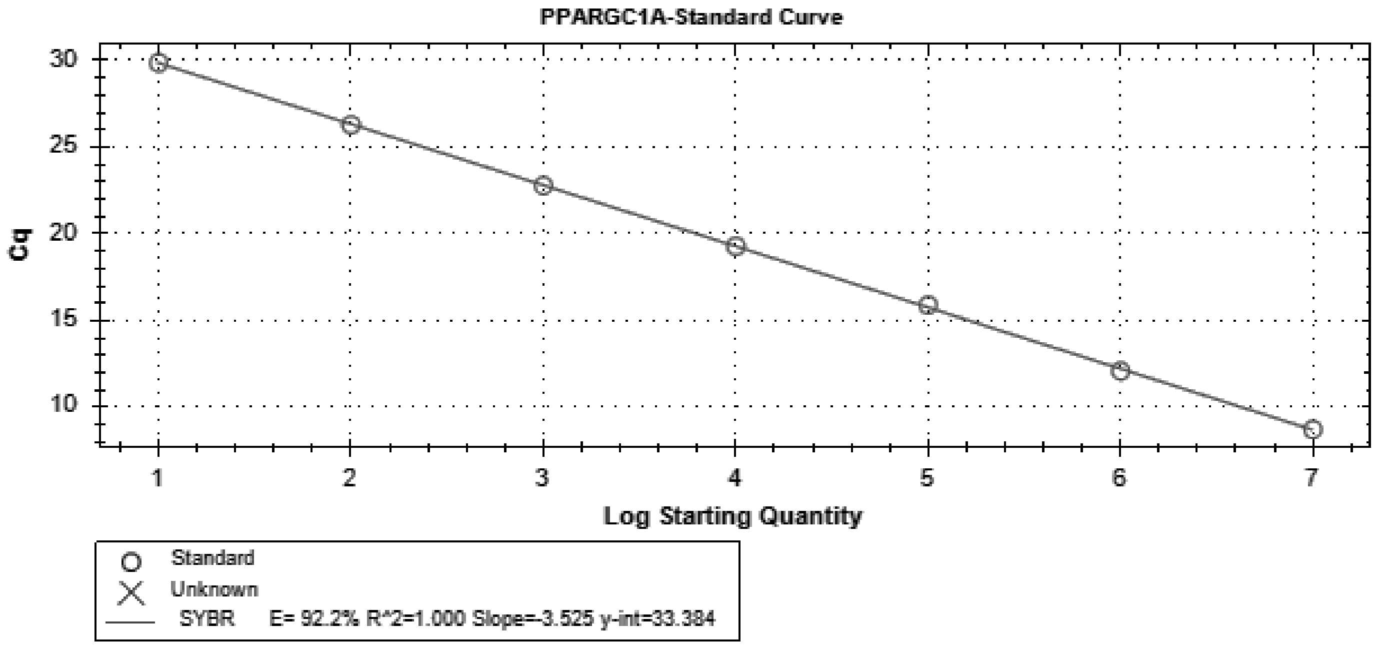
PPARGC1A-Standard Curve
Cq
Log Starting Quantity
Standard
Unknown
SYBR E= 92.2% R^2=1.000 Slope=-3.525 y-int=33.384

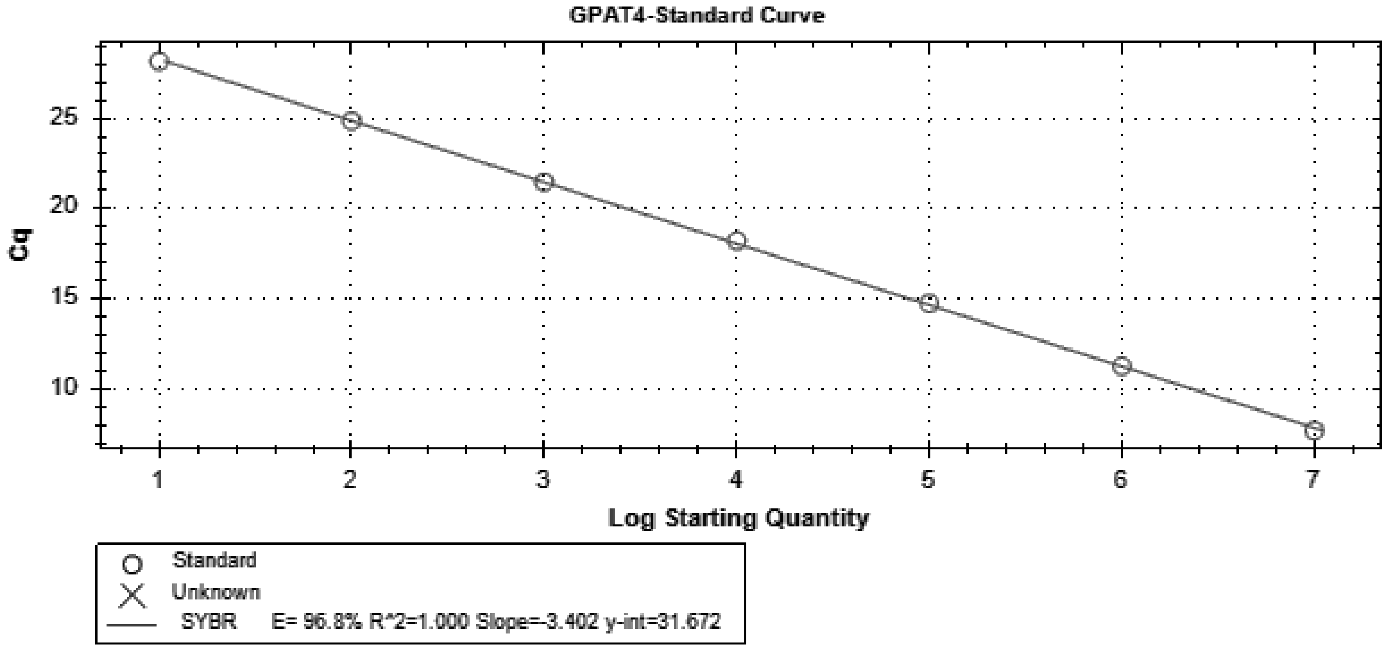
GPAT4-Standard Curve
Cq
Log Starting Quantity
Standard
Unknown
SYBR E= 96.8% R^2=1.000 Slope=-3.402 y-int=31.672
