# Supplementary material for: Using RNA-Seq to Identify Reference Genes of the Transition from Brown to White Adipose Tissue in Goats
Source: Animals (Basel). 2020 Sep 10;10(9):1626. doi: 10.3390/ani10091626 (PMC7552189; doi:10.3390/ani10091626)
Supplement: Supplementary file 1 [file animals-10-01626-s001.zip › Additional files/Table S2.docx]

**Table S2. Results of sample RNA quality determination**

| **Sample name** | **RIN-value** | **28S/18S** | **OD260/280** |
| --- | --- | --- | --- |
| D1-1 | 9.1 | 2.64 | 2.11 |
| D1-2 | 8.9 | 2.47 | 2.11 |
| D1-3 | 8.9 | 2.29 | 2.12 |
| D1-4 | 8.3 | 1.64 | 2.12 |
| D30-1 | 8.6 | 2.2 | 2.09 |
| D30-2 | 8.1 | 2.07 | 2.1 |
| D30-3 | 8.5 | 2.35 | 2.02 |
| D30-4 | 8.7 | 2.09 | 2.09 |
| Y1-1 | 8.3 | 2.47 | 2.1 |
| Y1-2 | 8.8 | 2.09 | 1.81 |
| Y1-3 | 9.1 | 2.21 | 2.05 |
| Y1-4 | 8.3 | 2.18 | 2.08 |
