# Supplementary figures and images for: Using RNA-Seq to Identify Reference Genes of the Transition from Brown to White Adipose Tissue in Goats
Source: Animals (Basel). 2020 Sep 10;10(9):1626. doi: 10.3390/ani10091626 (PMC7552189; doi:10.3390/ani10091626)

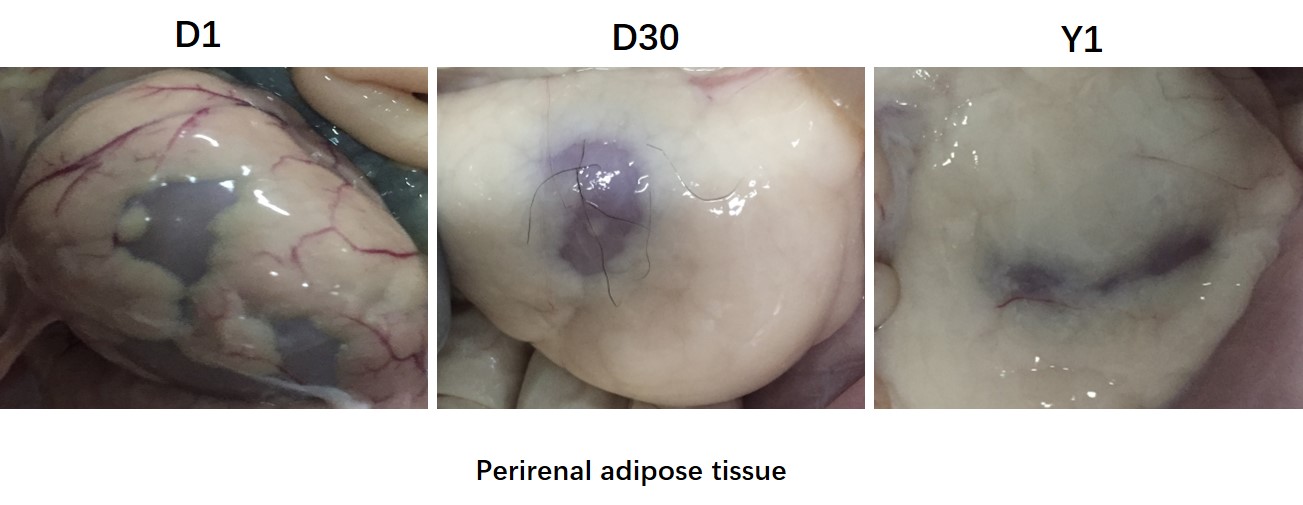

Supplement: Supplementary file 1 [file animals-10-01626-s001.zip › Additional files/Figure S1.jpg]

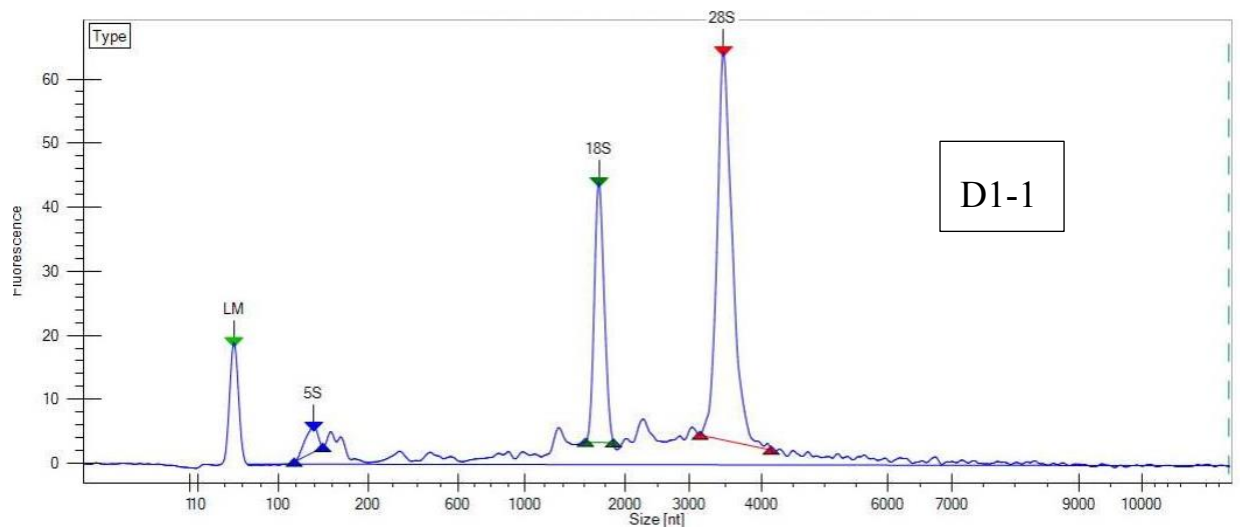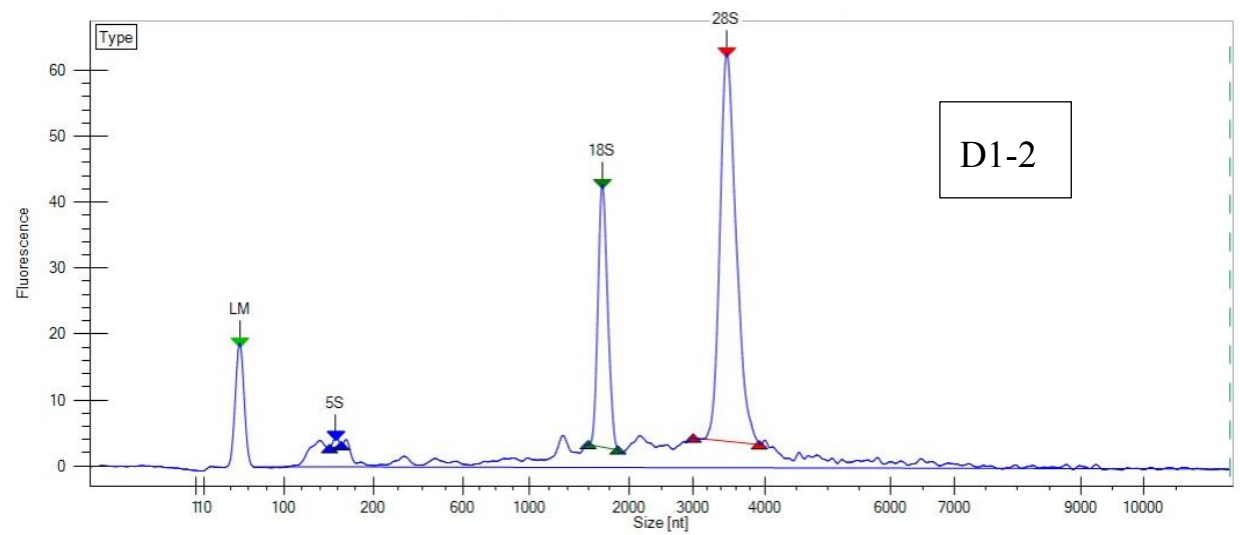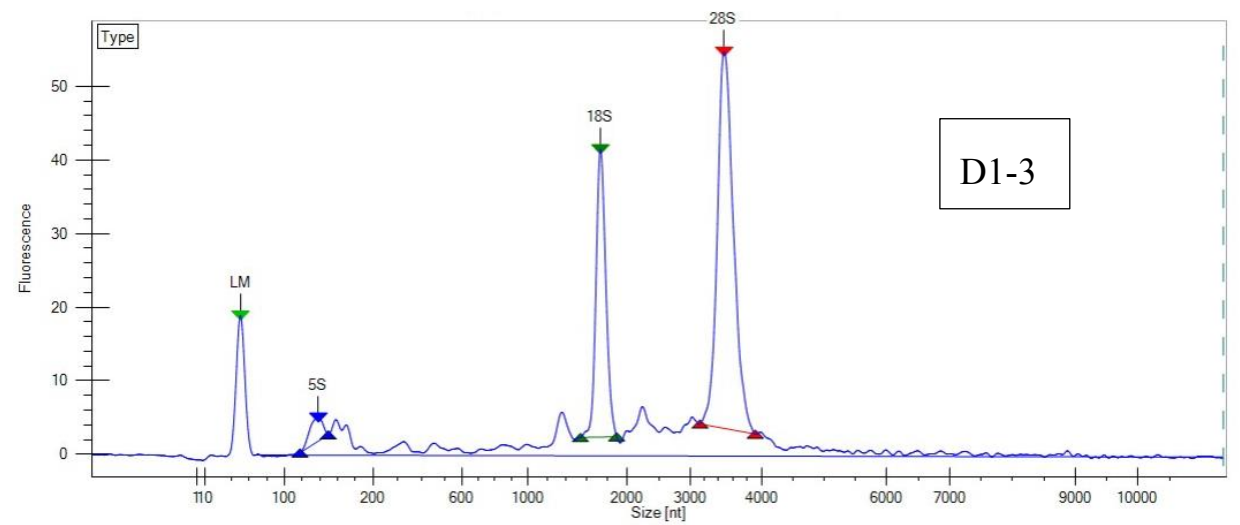

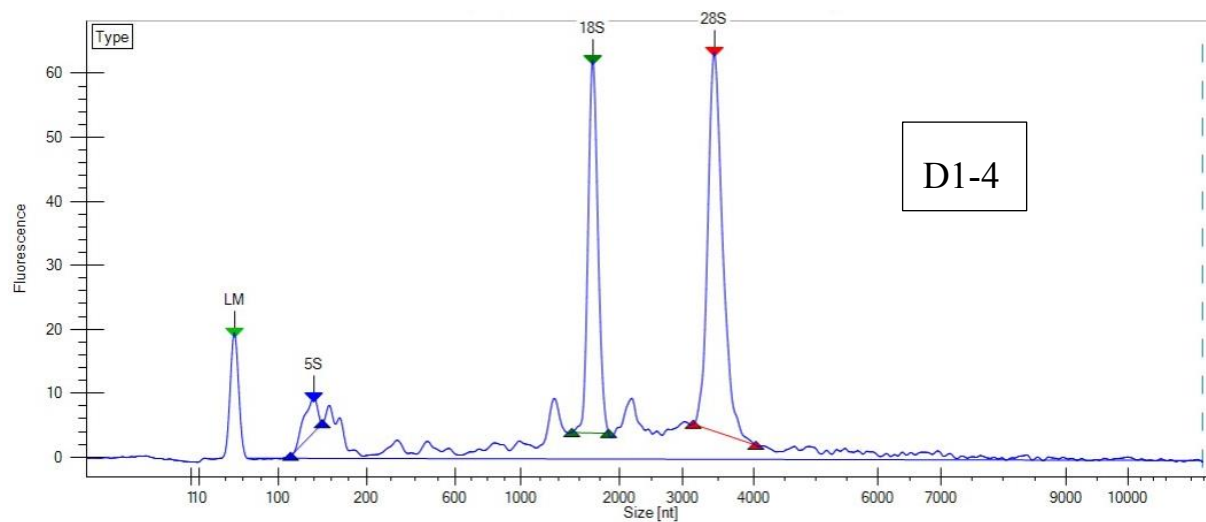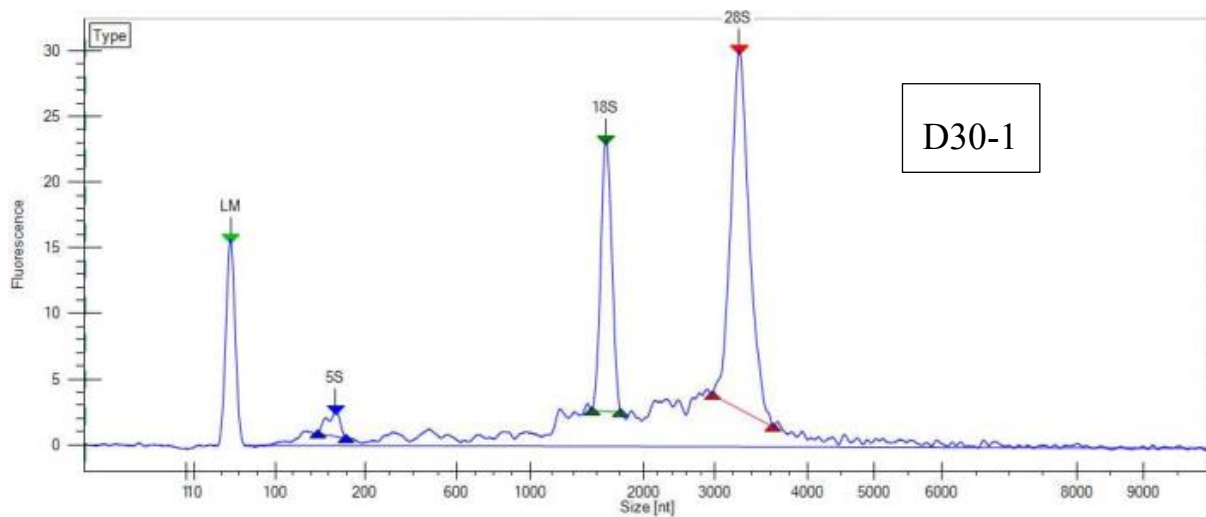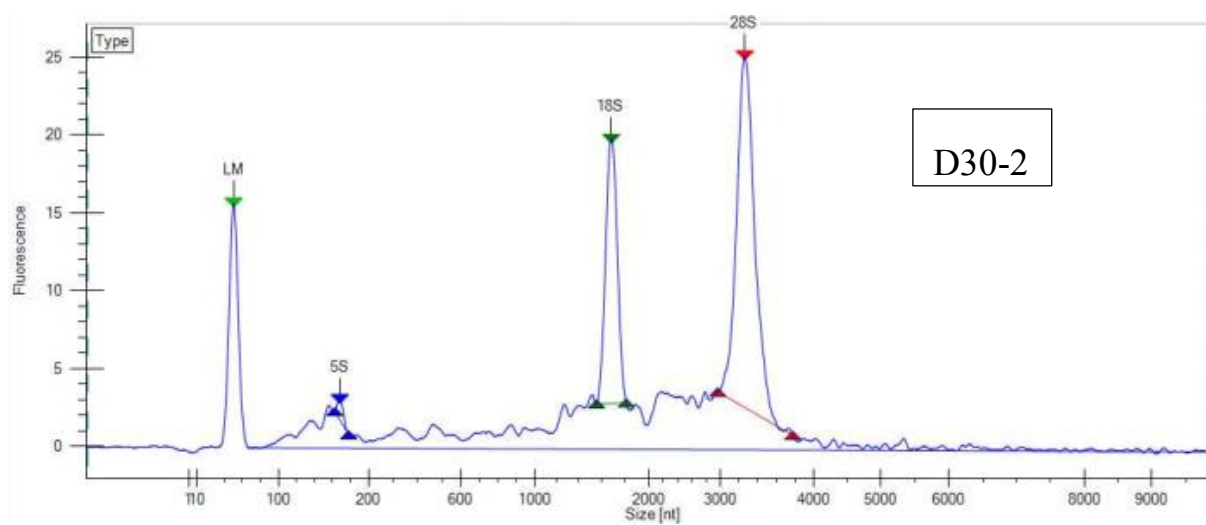

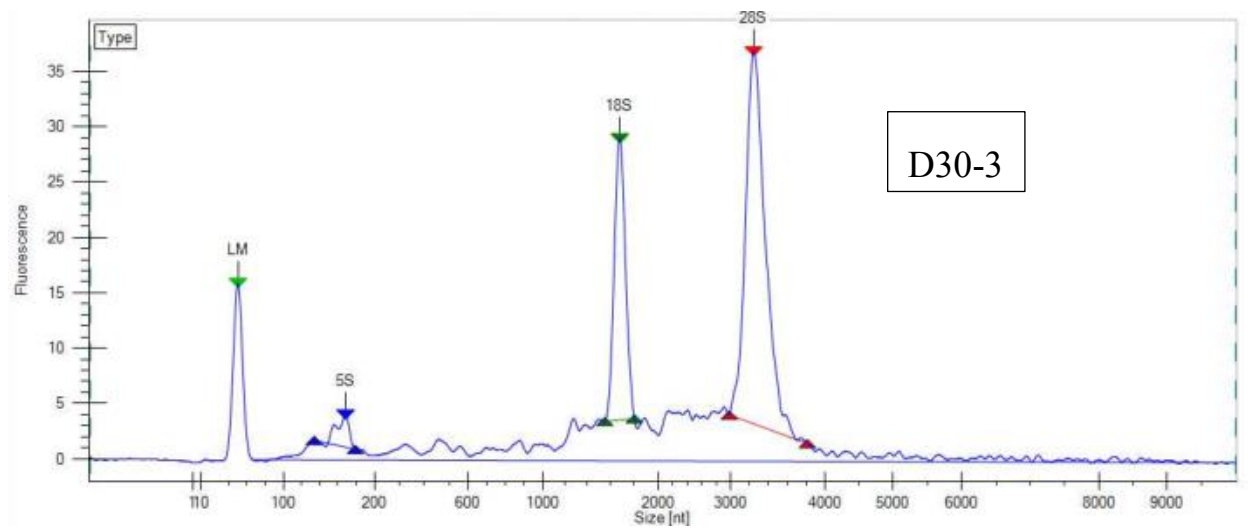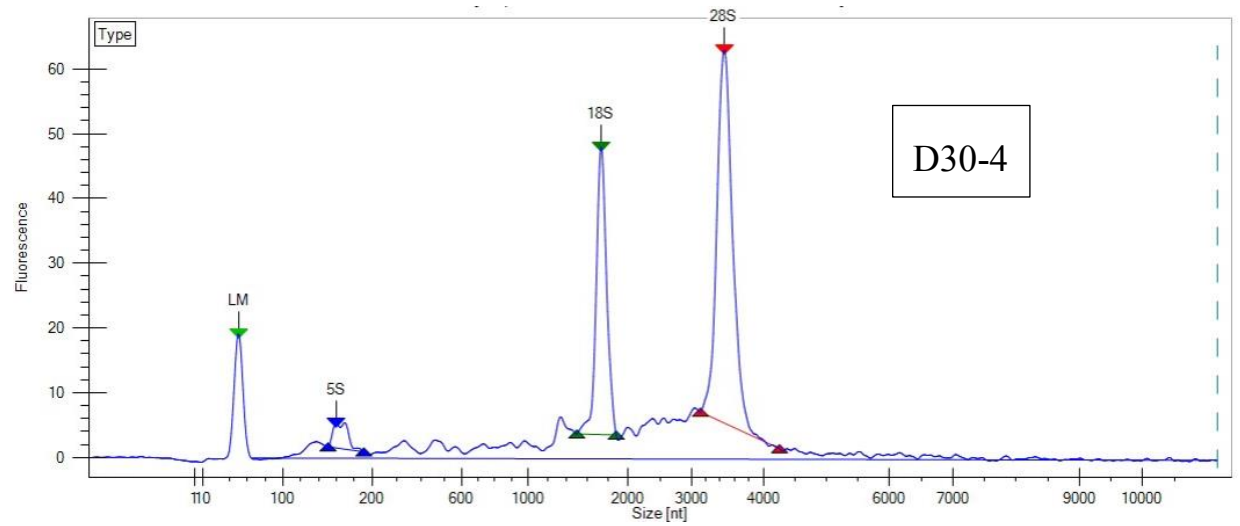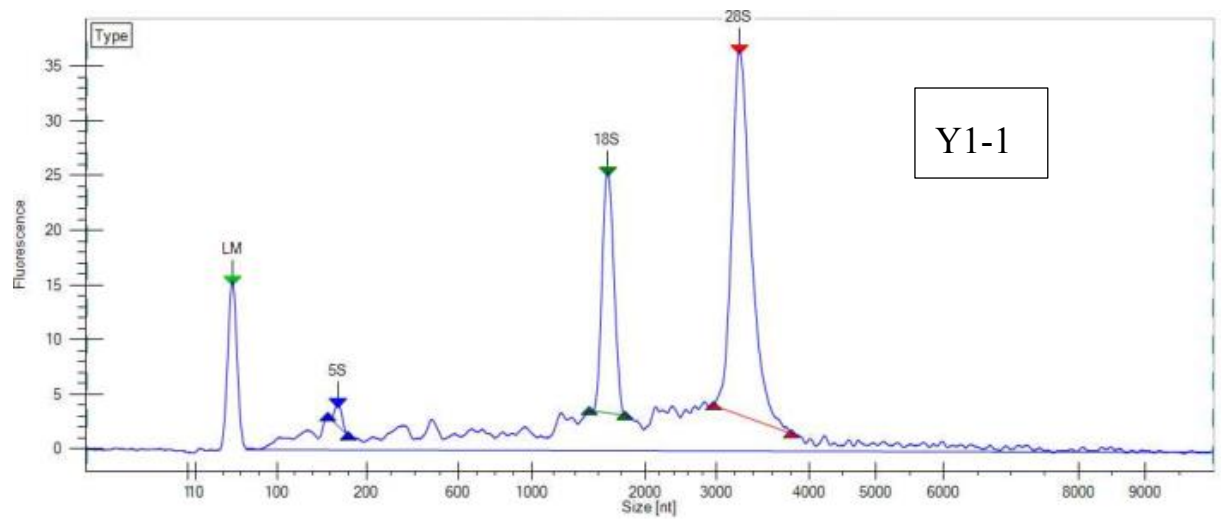

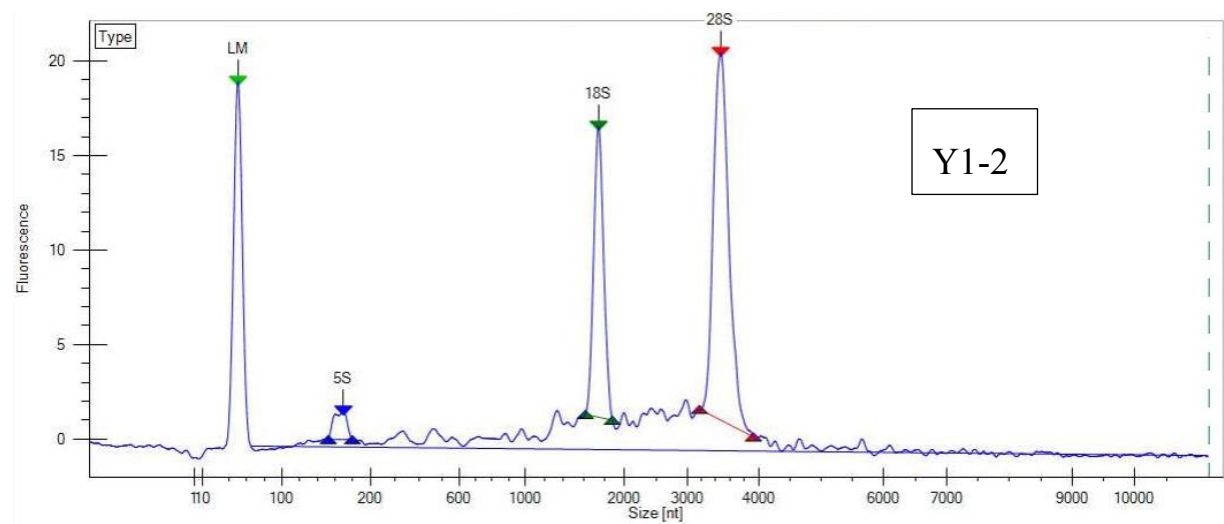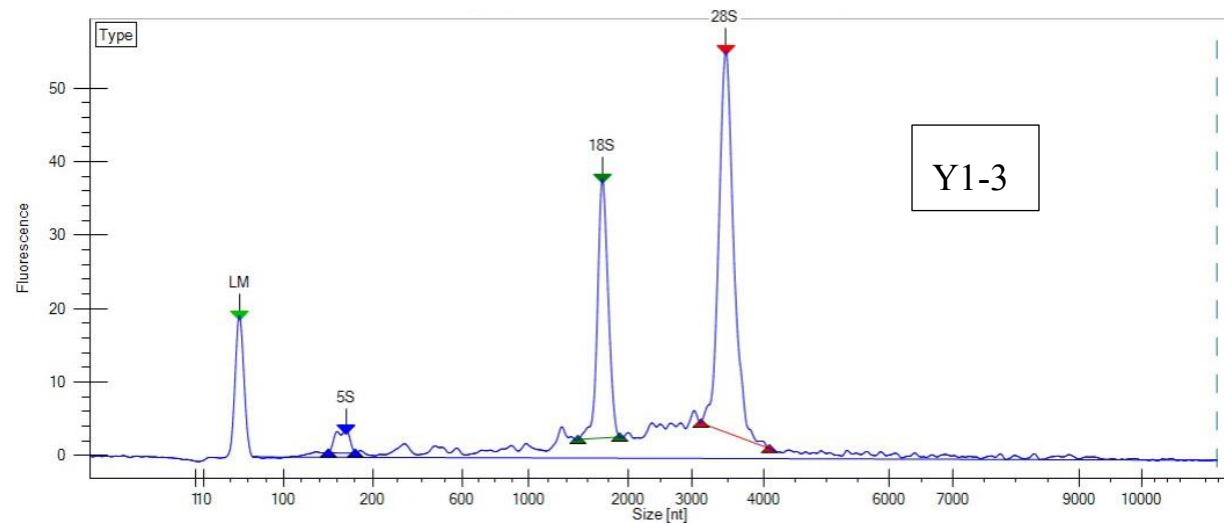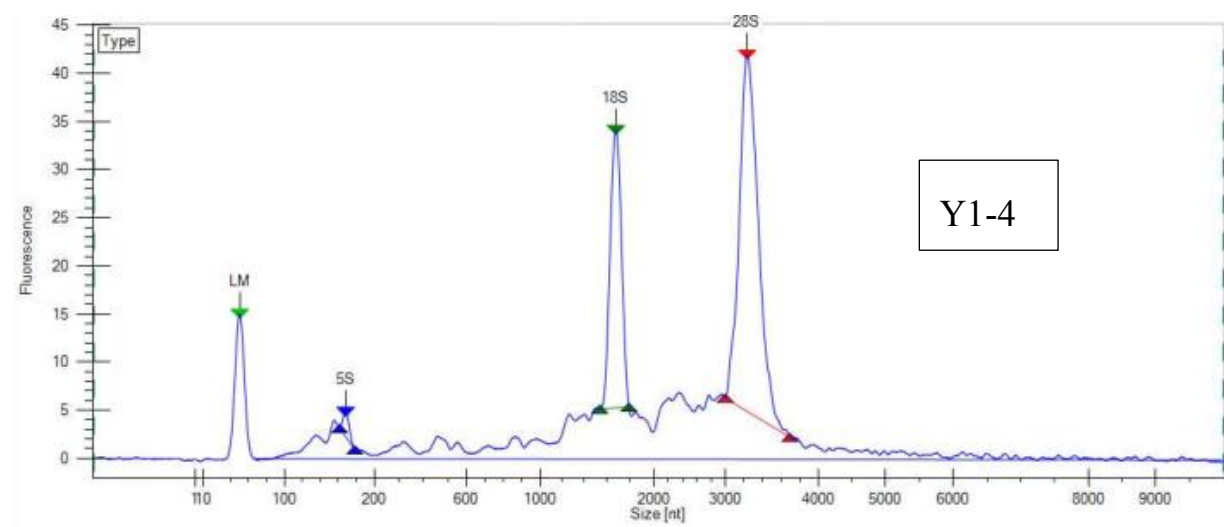

Supplement: Supplementary file 1 [file animals-10-01626-s001.zip › Additional files/Figure S2.pdf]

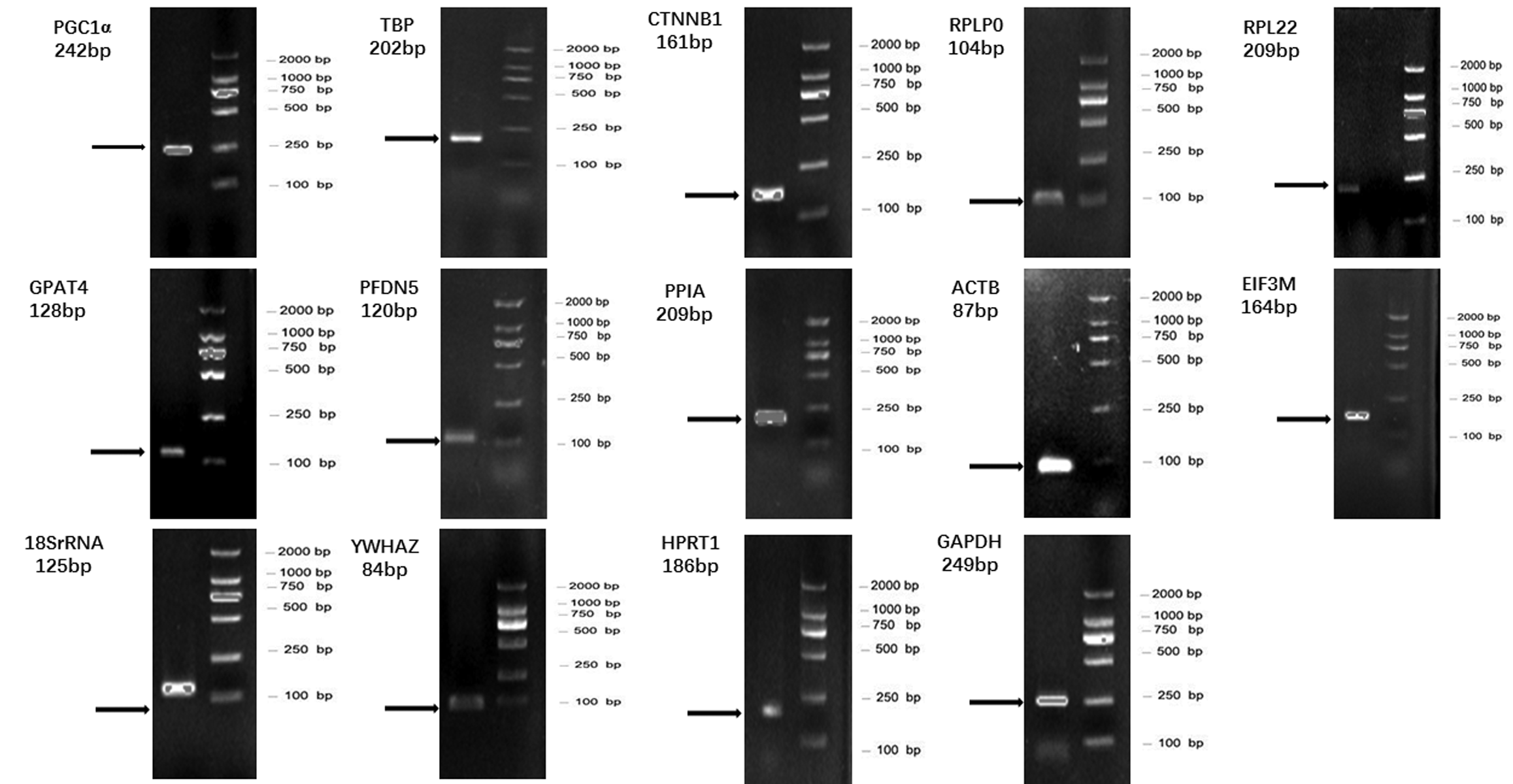

Supplement: Supplementary file 1 [file animals-10-01626-s001.zip › Additional files/Figure S3.tif]
